# Fluorescent Probes for Imaging in Humans: Where Are We Now?

**DOI:** 10.1021/acsnano.3c03564

**Published:** 2023-10-03

**Authors:** Deborah Seah, Zhiming Cheng, Marc Vendrell

**Affiliations:** † School of Chemistry, Chemical Engineering and Biotechnology, Nanyang Technological University Singapore 637371, Singapore; ‡ Centre for Inflammation Research, The University of Edinburgh, EH16 4UU Edinburgh, U.K.; § IRR Chemistry Hub, Institute for Regeneration and Repair, The University of Edinburgh, EH16 4UU Edinburgh, U.K.

**Keywords:** fluorescence-guided surgery, cancer, instrumentation, image analysis, diagnostics, dyes, translation, clinical trials

## Abstract

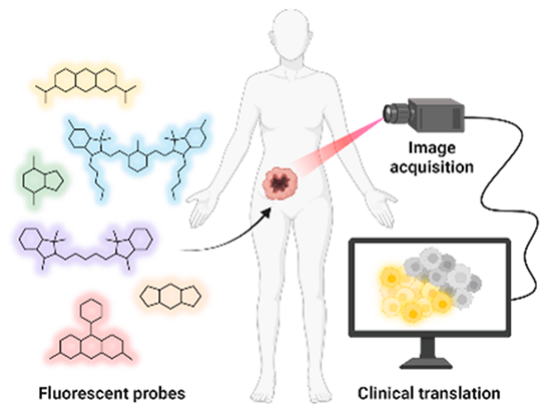

Optical imaging has become an indispensable technology in the clinic. The molecular design of cell-targeted and highly sensitive materials, the validation of specific disease biomarkers, and the rapid growth of clinically compatible instrumentation have altogether revolutionized the way we use optical imaging in clinical settings. One prime example is the application of cancer-targeted molecular imaging agents in both trials and routine clinical use to define the margins of tumors and to detect lesions that are “invisible” to the surgeons, leading to improved resection of malignant tissues without compromising viable structures. In this Perspective, we summarize some of the key research advances in chemistry, biology, and engineering that have accelerated the translation of optical imaging technologies for use in human patients. Finally, our paper comments on several research areas where further work will likely render the next generation of technologies for translational optical imaging.

Optical imaging is a noninvasive technology that employs light to interrogate biological systems and cellular function. Optical imaging is of growing relevance for clinical applications, as the interaction of light with various tissue components can allow clinicians to visualize abnormalities in tissue. For instance, fluorescence-guided intraoperative imaging can aid surgeons in accurately defining tumor margins for resection and assist in identifying lesions that may have been missed during visual inspection or palpation. Unlike nuclear imaging modalities such as positron emission tomography, optical imaging does not involve ionizing radiation, making it safer for patients. Furthermore, in contrast to other imaging modalities, the tunability of fluorophores can be used to obtain functional readouts and relevant molecular information to assist in clinical decision making. Despite its clear advantages, optical imaging is affected by the fact that light emitted by fluorescent agents can be absorbed by blood cells and the surrounding tissues, thus reducing the contrast and signal-to-noise ratios. To overcome this limitation, there has been growing interest in fluorophores that absorb and emit in the first and second near-infrared (NIR) windows of 700–1000 and 1000–1700 nm respectively, as well as imaging devices that can detect such signals.

Even though significant advancements have been achieved in the field of optical imaging, only a few chemical probes have been translated to the clinic. The slow progress of clinical translation can be attributed to three main areas. (1) Fluorophore development: fluorophores should be bright and display high signal-to-background ratios and limited photobleaching. To achieve this, there has been growing interest in fluorophores in the NIR region. However, it is difficult to develop molecular fluorophores with long-wavelength emission and high brightness and there are only a few NIR fluorophores for use in clinical imaging. (2) Selection of target: optical imaging agents must bind selectively to the targeted ligands in diseased tissue (e.g., cancer-associated receptors) to generate sufficient contrast at the target area. Currently, the two most common classes of biological targets studied are cancer-associated surface receptors and enzymes. Other targeting moieties will require further characterization and clinical assessment to verify their correlation with disease occurrence and progression. (3) Development of hardware and software: imaging devices used in the clinic should be sensitive, able to detect NIR wavelengths, and have ergonomic features for ease of use in the clinic. As the most common NIR fluorophore used in the clinic is indocyanine green (ICG), most of the clinical imaging instrumentation considers the excitation and emission wavelengths of ICG, with fewer imaging devices being compatible with other fluorophores.

Currently, there is a diverse range of chemical approaches and biological targets for translational optical imaging. Excluding nanoparticles and other drug delivery systems (e.g., liposomes, micelles), chemical agents currently in use for translational studies in humans can be classified on the basis of their size, namely small-molecule-based, peptide-based, and antibody-based imaging agents. In contrast to initial work done with nonspecific agents like ICG, there is particular interest in the design of targeted molecular agents, which can respond to the expression of cell surface receptors or to the activity levels of enzymes. At the same time, the advancement of imaging systems is vital to ensure effective translation of molecular optical agents to the clinic. Current engineering solutions for optical imaging in humans range from standalone fluorescence imaging systems to hand-held devices or goggles for surgeons, with recent prototypes aiming at increasing sensitivity, detecting longer wavelengths, and exhibiting ergonomic display and usage features.

In this Perspective, we present an overview of recent advances in translational optical imaging in humans from the last 10 years. We first review the chemical approaches in developing molecular imaging agents, followed by a discussion on the biological targets reported for optical imaging and the various engineering solutions for imaging in humans in the clinic. Through this Perspective, we also provide an outlook on the possible future approaches for imaging in humans and discuss the avenues in which further research and development would accelerate the translation of optical imaging from bench to bedside.

## 1 Chemical Development of Molecular Probes for Imaging in Humans

Molecular imaging probes for translational optical imaging vary largely in size, with the smallest being small-molecule imaging agents and the largest typically being antibody-based constructs. The size of the probes can influence the mode of administration. Small probes tend to be more hydrophobic and thus are locally or topically applied, whereas larger probes, such as antibody-based conjugates, usually require systemic injection well in advance of image acquisition because they are too large to permeate within deep layers of tissues.^1^ Furthermore, the size of the probes is one of the main determinants for retention time within the vascular and extravascular compartments in the body.^2^ Small renal clearable probes display shorter retention times, resulting in decreased target accumulation and reduced tumor-to-background signals. Current strategies to increase the retention time include biodegradable probes consisting of “soft” materials with flexible shapes, such as proteins and polymers. Such probes can be degraded into smaller fragments *in vivo* with concomitant renal clearance.^2^

**Figure 1:**
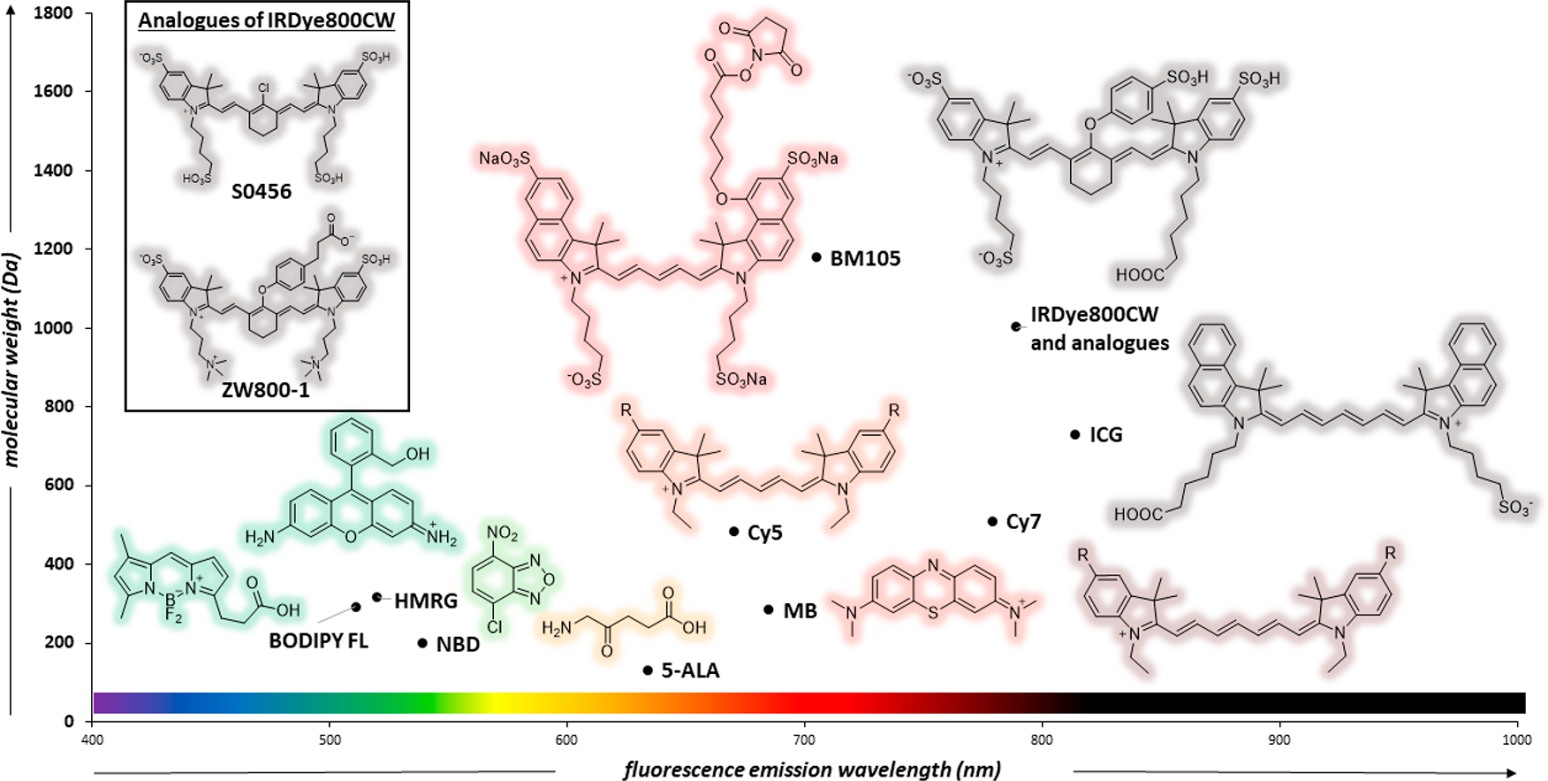
Fluorophores used for human clinical imaging. The chart displays molecular weight (*y*-axis) and emission wavelength maxima (*x*-axis) and includes BODIPY-FL (511 nm, 292 Da), hydroxymethyl rhodamine green (HMRG, 520 nm, 317 Da), nitrobenzoxadiazole (NBD, 539 nm, 200 Da), 5-aminolevulinic acid (5-ALA, 131 Da) as the nonfluorescent precursor of the fluorescent PpIX (634 nm), Cy5 (670 nm, 484 Da), MB (685 nm, 284 Da) and BM105 (705 nm, 1180 Da). NIR fluorophores include Cy7 (779 nm, 510 Da), IRDye800CW (789 nm, 1002 Da), and ICG (814 nm, 731 Da).

Another important factor in the design of chemical agents for clinical translation is the photophysical properties of the fluorophores. Fluorophores commonly used for clinically translated imaging in humans have been summarized in Figure 1, based on their emission wavelengths and molecular weights. Currently, FDA-approved probes include ICG, methylene blue (MB), and 5-aminolevulinic acid (5-ALA) as the precursor for protoporphyrin IX (PpIX). MB and PpIX absorb and emit in the visible light region with limited resolution over autofluorescence and penetration depth. As such, there has been growing interest in fluorophores that emit in the NIR-I window where cellular components exhibit decreased photon scattering, autofluorescence, and light absorption. Furthermore, probes that absorb in the NIR region require photons of lower energy, increasing the applicable laser power (due to increased maximum permissible exposure), therefore improving image quality.^2^ Future probes for clinical use may incorporate alternative fluorophores. For instance, highly photostable fluorescent scaffolds such as BODIPYs and oxazines have been already employed in preclinical studies and may undergo clinical trials in the near future. While some of these fluorophores may not be active within the NIR spectrum, they could still find use in a range of clinical applications such as those employing local administration. FDA-approved ICG and MB depend on the enhanced permeability and retention (EPR) effect to accumulate in tumors, which is often insufficient to obtain high signal-to-background ratios.^3^ To counterbalance this effect, various ligand-based targeting probes, such as peptide- and antibody-based probes, have been developed to increase the accumulation in the target. In this section, we review the main different types of molecular imaging probes that have been used for imaging in humans (Table 1).

**Table 1:** Summary of Optical Agents Reported in Human Clinical Imaging Trials

optical imaging agent	condition	trial number	clinical stage
methylene blue	small intestine neuroendocrine tumors	NL9305^a^	
5-ALA	glioma	NCT01116661	phase 2
OTL-38	ovarian cancer	NCT02317705	phase 2
	neoplasms	NCT02602119	phase 1
	ovarian cancer	2013-004774-10^b^	phase 1
		2014-002352-12^b^	
	neoplasms, pituitary neoplasms	NCT02629549	phase 1
EM-137	Barrett’s esophagus, esophageal cancer, dysplasia in Barrett’s esophagus	NCT03205501	phase 1
cRGD-ZW800-1	colon cancer	2017-002772^b^	phase 1 and phase 2
	respiratory infections	NCT02491164	phase 1
AVB-620	breast cancer	NCT02391194	phase 1
cetuximab-IRDye800CW	head and neck cancer	NCT01987375	phase 1
panitumumab-IRDye800CW	pancreatic adenocarcinoma	NCT03384238	phase 1 and phase 2
LUM015	sarcoma, soft tissue sarcoma, breast cancer	NCT01626066	Phase 1
VGT-309	cathepsin activity in human pulmonary tumors	ACTRN12621000301864^c^	phase 1 and phase 2
PARPi-FL	oral squamous cell carcinoma	NCT03085147	phase 1 and phase 2
folate-FITC	ovarian cancer	NTR1980^d^	
		EudraCT 2009-010559-29^b^	
benvazucimab-800CW	breast cancer	NCT02583568	phase 1 and phase 2
colon KCC heptapeptide	colon polyps, colorectal cancer, inflammatory bowel disease (IBD)	NCT02156557	phase 1
FITC-adalimumab	Crohn’s disease	NCT01275508	phase 1 and phase 2

a ICTRP registration code.

b European Clinical Trials Database.

c Australian New Zealand Clinical Trials Registry.

d Dutch Trial Register.

### Small-Molecule Imaging Agents

Currently, three small-molecule fluorophores have been approved by the FDA as diagnostic agents for use in clinical practice: ICG, MB, and 5-ALA. ICG is an NIR tricarbocyanine dye that is excited around 760–785 nm and emits around 820–840 nm.^4^ As there are no functional groups available for conjugation to targeting moieties, ICG is a nonspecific contrast agent and depends on the EPR effect for accumulation in tumors. For instance, intraoperative peritumoral injection of ICG has been used for sentinel lymph node (SLN) mapping in lung cancer with a sensitivity of 87.5% and negative predictive rate of 100%.^5^ On the other hand, MB is a clinically available tracer that absorbs around 665 nm and emits around 685 nm. When intravenously injected, MB can accumulate in neuroendocrine tumors; a clinical study to investigate the detection of small intestinal neuroendocrine tumors (SI-NETs) with MB found that 93% of lesions had a positive fluorescence signal and MB fluorescence enabled the identification of additional metastases in 3 out of 16 patients (ICTRP NL9305).^6^ 5-ALA is a porphyrin precursor that, when supplied exogenously, is consumed by certain cancers to form the fluorescent PpIX, resulting in tumor-specific fluorescence.^7^ The fluorescence of PpIX allowed for the detection of focal intratumoral areas of malignant transformation in 22 patients with suspected diffusely infiltrating gliomas (NCT01116661). Additional quantitative analysis of PpIX aided visualization of low-grade glioma tissue that is typically undetected by conventional fluorescence.^8^

However, unbound tracers, such as ICG, MB, and 5-ALA, in systemic circulation can lead to nonspecific background fluorescence and decreased signal-to-background ratios that hamper the visualization of specific biological targets. It is thus important to establish a biological basis for imaging contrast with sufficient target-to-background ratios *in vivo*. One example of a targeted small-molecule agent currently undergoing clinical trials is OTL-38. OTL-38 is a folate receptor α (FRα) targeting agent comprising a folate moiety that is conjugated to the NIR dye S0456. S0456 is a derivative of IRDye800CW, in which the phenoxyether bridge is replaced by a chloride atom and an additional sulfonate group provides increased water solubility.^3^ S0456 shows an absorption maximum around 776 nm and emits between 790 and 805 nm with maximum emission at 796 nm.^9^ OTL-38 was modified from the folate-fluorescein isothiocyanate (FITC) conjugate EC-17^10^ that emits in the visible range and whose signals can be hard to distinguish from tissue autofluorescence. OTL-38 enables tumor visualization with high signal-to-background ratios as FRα is overexpressed in tumors and nonexpressing tissues clear OTL-38 quickly. In 2019, a phase II multicenter, open-label trial of OTL-38 injection for the intraoperative imaging of FRα positive ovarian cancer demonstrated a sensitivity of at least 85% with acceptable toxicity (NCT02317705).^11^ Other successful clinical trials include the imaging of endometrial cancer,^12^ pulmonary adenocarcinomas^13^ (NCT02602119) and pituitary adenomas.^14^ While less specific than larger macromolecules such as peptides and antibodies, small-molecule imaging probes have the advantage of being more rapidly cleared from the body,^15^ which can result in optimal target-to-background ratios at shorter and more manageable time points.

### Peptide-Based Molecular Imaging Agents

Peptide-based probes can be categorized into three broad classes, namely, targeting probes, enzyme-activated imaging probes, and cross-linking probes. Targeting probes contain a peptide moiety for biomolecule recognition, with binding typically being highly selective and noncovalent. The peptide sequence drives binding to the biological target, resulting in the accumulation of the fluorophore at the target site. However, the design of such probes is limited to ligands with a strong binding affinity. Such probes include the c-Met targeted EMI-137,^16,17^ the integrin-targeted cRGD-ZW800-1,^18^ and the recently reported NBD-PMX probe for binding lipid A in Gram-negative bacteria.^19^

During the construction of these probes, different methods were used to identify the desired peptide sequence. The water-soluble probe EMI-137 comprises a sulfonated Cy5-tagged 26 amino acid long cyclic peptide with two disulfide bridges and targets the transmembrane human growth factor receptor c-Met.^16,17^ The amino acid sequence was selected from a M-13 phage display library and was chosen for its ability to bind to c-Met without affecting downstream signaling pathways. Fluorescence molecular endoscopy in 15 Barrett’s neoplasia patients with EMI-137 found that different topical and intravenous doses of EMI-137 appeared to be safe and able to identify 16 out of 18 lesions, with modest target-to-background signals (NCT03205501).^17^ On the other hand, integrin-targeted cRGD-ZW800-1 utilizes the well-studied RGD pentapeptide sequence Arg-Gly-Asp-tyr-Lys.^18^ The cyclic cRGD was conjugated to the zwitterionic NIR fluorophore ZW800-1 for tumor imaging. Due to its overall zwitterionic chemical structure and neutral charge, cRGD-ZW800-1 has less nonspecific uptake and thus higher signal-to-background signals.^20^ A phase II study found that cRGD-ZW800-1 exhibited renal-predominant clearance, providing rapid elimination from the body and nonspecific fluorescence in the bowel, improving the quality of images (European Trials Database 2017-002772-60).^18^ Finally, the topical fluorescent probe NBD-PMX was reported as a probe for lipid A in Gram-negative bacteria (NCT02491164).^19^ Polymyxins (PMXs) are amphipathic, cyclic antimicrobial peptides that bind lipid A on the outer membrane of Gram-negative bacteria. The eventual sequence of PMX was selected from a panel of modified PMX constructs, based on the binding specificity to *Pseudomonas aeruginosa* vs *Staphylococcus aureus* as Gram-negative and Gram-positive bacteria, respectively. PMX was linked to the environmentally sensitive fluorophore nitrobenzoxadiazole (NBD) to generate fluorescence emission upon engagement with the bacterial membranes and was topically administered to the lungs of human patients.

Enzyme-activated imaging probes typically consist of peptide sequences that are recognized by the enzymatic targets. Such probes are designed to have a negligible fluorescence signal in the intact state, while their fluorescence signals are amplified upon cleavage by the target enzyme. Common enzymatic targets include cathepsins and matrix metalloproteinases (MMPs). This class of probes typically exploits the Forster Resonance Energy Transfer (FRET) effect, where cleavage of the peptide linker between a fluorophore and quencher releases the quencher and allows the fluorescence signal to be detected. One successful clinical translation is the cathepsin-activated probe LUM015. LUM015 is a PEGylated protease-activated far-red imaging probe that combines Cy5 (fluorophore), QSY21 (quencher), the cathepsin-substrate Gly-Gly-Arg-Lys sequence, and a 20 kDa PEG chain.^21^ Proteolytic cleavage by cathepsins releases optically active fragments containing Cy5, which can be readily detected. The fragment containing Cy5 and no PEG was found to be the major fragment that correlated with the tumor presence in humans. In human tumor samples, the fraction of activated probe in tumors was 0.26, which is 60% higher than that in healthy tissues. Other than cathepsin-targeted LUM015, the clinical translation of ratiometric MMP-activity targeted probe AVB-620 has been reported (NCT02391194).^22,23^ AVB-620 comprises two fluorophores, Cy5 and Cy7, linked by the peptide sequence Pro-Leu-Gly-Cys(Me)-Ala-Gly, which is cleaved by MMP2 and MMP9 and leads to changes in the Cy7/Cy5 fluorescence ratios. This allows for simultaneous ratiometric detection, decreasing artifactual signal variations, and improving imaging accuracy.

Cross-linking probes contain peptide-based binding regions that are recognized by the target proteins. Upon binding, the chemical reactive group reacts with a specific residue in the binding pocket to form an irreversible covalent linkage. The cathepsin activity-based probe VGT-309 utilizes ICG as its fluorophore and the IRDye QC-1 as the quencher.^24^ ICG was chosen as the fluorophore, as it can be detected by FDA-approved surgical imaging devices. Irreversible covalent binding of the phenoxymethylketone electrophile in VGT-309 releases the quencher, allowing an NIR fluorescence signal from ICG to be detected with notable tumor-to-background ratios. VGT-309 was also found to localize in visually occult, nonpalpable tumors in real time, indicating the potential application of VGT-309 in fluorescence-guided tumor resection. Furthermore, designing cross-linking probes allows low-affinity ligands to be turned into useful targeting ligands due to the addition of a chemically reactive group to the binding sequence.

However, cathepsin- and MMP-activatable probes target endopeptidases and, thus, must be administered intravenously at high doses. Conversely, topically administered probes such as gGlu-HMRG can be administered at lower doses.^25^ gGlu-HMRG is an activity-based fluorophore that becomes fluorescent upon hydrolysis by γ-glutamyltranspeptidase (GGT) due to intramolecular spirocyclization and release of HMRG.^26^ Fluorescent HMRG is able to permeate the lipid bilayer of the plasma membrane to enter cancer cells and accumulate in the lysosome. gGlu-HMRG was evaluated *ex vivo* in patients with suspected or biopsy-proven oral cancer. Further developments for gGlu-HMRG will involve clinical trials investigating *in vivo* usage. Compared with affinity-based probes, peptide-based activatable probes have the advantage that the unbound circulating fraction emits no fluorescence signals, which can lead to enhanced sensitivity. Imaging probes with low-molecular-weight peptides tend to have more favorable pharmacokinetics and tissue distribution patterns than antibody-based probes. They are more permeable and less likely to elicit an immunogenic response. Furthermore, they are easily synthesized and chemically modified and can thus be produced in large quantities at a low cost.

### Antibody-Based Probes

Similar to the peptide-based imaging probes, antibodies establish a biological basis for generating contrast with a sufficient target-to-background ratio *in vivo*. Antibody-based probes undergoing clinical trials include various antibody conjugates with IRDye800CW. IRDye800CW is a NIR fluorescent dye that is commonly used for labeling antibodies, as the conjugation with IRDye800CW does not affect the binding capacity, pharmacokinetics, or biodistribution of the antibody of choice. Successful clinical trials include cetuximab-IRDye800CW (NCT01987375)^27^ and panitumumab-IRDye800CW (NCT03384238)^28^ for head and neck cancer, anti-HER2 VHHS and IRDye800CW conjugate for breast cancer,^29^ and anti-GD2-IRDye800CW for imaging of neuroblastoma.^30^ It has been reported that, for cetuximab-IRDye800CW and panitumumab-IRDye800CW, the toxicity and pharmacodynamic profiles of the antibody-IRDye800CW conjugates match those of the parent antibody compound.^31^ As such, other therapeutic antibodies can be repurposed as imaging agents. In addition to IRDye800CW-antibody conjugates, SGM101 is another well-established antibody-based molecular imaging probe. SGM101 is a carcinoembryonic antigen (CEA) targeting probe for the intraoperative detection of primary pancreatic ductal adenocarcinoma (PDAC)^32,33^ and lung adenocarcinomas.^34^ It consists of a CEA-specific chimeric antibody conjugated to the NIR fluorescent dye BM105. BM105 is a fluorophore specifically developed to minimize aggregation during the coupling reaction with the anti-CEA chimeric mAb.^35^ Consequently, the choice of BM105 improved the optical properties of SGM101. Monoclonal antibodies and their genetically engineered fragments provide a good platform for designing highly specific molecular imaging probes due to their superior binding specificity. The binding sites can also be modified to target a broad variety of cell surface epitopes, allowing for versatility as imaging tools. However, an inherent limitation of antibody–dye conjugates is their slow pharmacokinetics. Antibody-based probes exhibit prolonged circulating half-lives and slow clearance from the body, resulting in limited tumor-to-background signals at short time points.^36^ This shortcoming can be compensated for with the administration of antibody-based imaging agents several hours before imaging. Another area of concern for fluorescently tagged antibodies is the potential to cause immunogenic reactions, and when choosing the antibody for targeting, potential immunogenicity needs to be considered.

## 2 Choosing the Biological Target

Many studies translating optical imaging in humans have considered biological targets associated with cancer. Cancer is one of the leading causes of death worldwide, and surgical interventions are often the main treatments for cancer patients. As the likelihood for recurrence is related to the remaining tumor cells left behind during surgery, optical imaging has been considered as a technology to assist surgeons in defining tumor margins and detect lesions that might be missed by visual inspection or palpation.

Initial attempts at implementing optical imaging into clinical practice have employed fluorophores (e.g., MB and ICG) with no specific targeting features but with regulatory approval and good safety profiles in humans. For instance, one feasibility study evaluated intravenous injections of MB during breast-conserving surgery to facilitate intraoperative localization of the tumor tissue.^37^ Overall breast cancer identification rates using MB reached 83% (from a total of 24 patients), although no significant relationships were found between MB staining and receptor status or tumor grades, possibly due to the lack of molecular targeting. A follow-up MB study was run on 13 patients diagnosed with primary hyperparathyroidism and undergoing parathyroidectomy and assessed the use of MB to identify diseased and normal parathyroid glands.^38^ The intravenous injection of MB identified both parathyroid adenomas and normal parathyroid glands, suggesting potential use in patients where the identification of the parathyroid adenoma was difficult or when normal glands had to be identified. Similar studies—reviewed elsewhere—^39,40^ have also exploited the nonspecific targeting nature of ICG under different intraoperative cancer conditions, including colorectal, liver, or pancreatic surgery.

**Figure 2:**
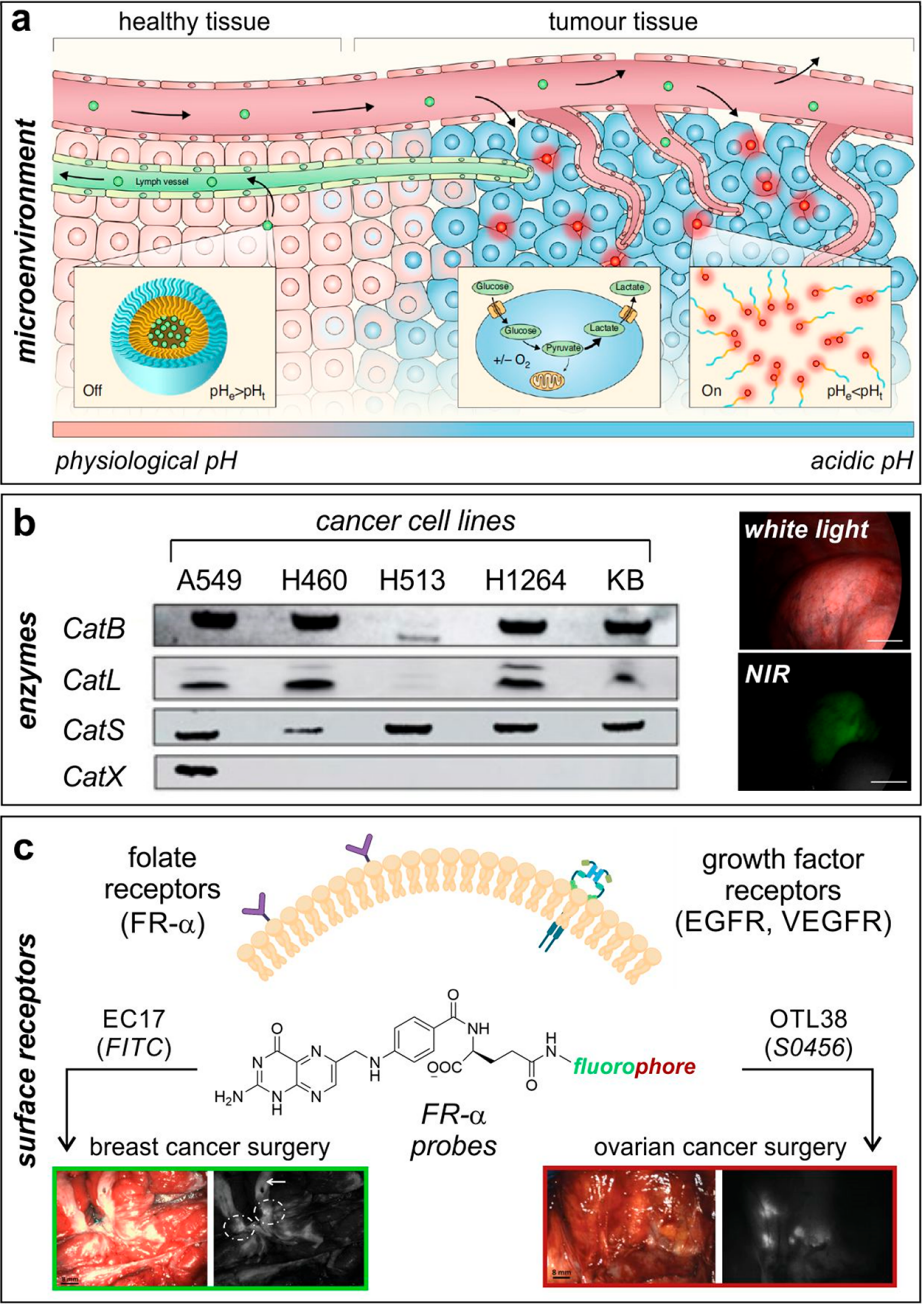
Biological targets for translational fluorescence imaging in humans. (a) Extracellular cancer acidosis as a generic target for a broad range of solid tumors.^41^ The tumor microenvironment turns acidic when the cancerous tissue becomes invasive; this is exploited for the ONM100 nanoparticles to extravasate because of enhanced permeability of the tumor vasculature and results in ONM-100 accumulation within the acidic extracellular matrix with a concomitant switch from the “off” (green) to the “on” (red) state. Reproduced under a Creative Commons license from ref (41). (b) Cathepsin-activatable agents for in-human imaging of cancer cells.^24^ Western blot analysis of cathepsin expression in human nonsmall lung cancer cells using KB human cervical carcinoma cells as a positive control. Representative white light (top) and NIR fluorescence (bottom) images of a pulmonary tumor after administration of the cathepsin-activatable VGT-309 probe. Scale bars: 1 cm. Reproduced from ref (24) with permission from the American Association for Cancer Research. (c) FR-α and growth factor receptors as potential biological targets for imaging of cancer tissue. (Left) FR-α probe EC17 (fluorophore: FITC) was employed during breast cancer surgery to identify a bisected primary breast cancer lesion by using fluorescence imaging (dashed circles). The white arrow indicates tissue autofluorescence signals.^48^ Reproduced under a Creative Commons license from ref (48). (Right) the FR-α probe OTL38 (fluorophore: S0456) was employed during ovarian cancer surgery to identify retroperitoneal lymph nodes containing metastases of ovarian cancer.^49^ Reproduced from ref (49) with permission from the American Association for Cancer Research.

In recent years, most efforts in translational optical imaging of cancer have focused on targeted approaches. Multiple biological targets have been considered, with cancer-associated cell surface receptors and enzymes being among the two most common classes. However, one shortcoming is their potentially limited applicability given the variability in receptor and enzyme expression among cancer patients (e.g., triple-negative breast cancer patients). To address this limitation, Sumer and van Dam decided to exploit extracellular cancer acidosis as a generic target that could find applicability for the detection of a broad range of solid tumors (Figure 2a).^41^ Even though tumor pH values depend on a number of factors (e.g., glucose and oxygen levels, vasculature) and can vary between 5.8 and 7.4, the authors developed ONM-100 as an ICG-containing pH-sensitive amphiphilic polymer that dissociated and fluoresced in the acidic extracellular tumor microenvironment. ONM-100 enabled detection of tumor-positive resection margins and lesions missed after palpation or visual inspection (total 30 patients), highlighting the fact that physiologic parameters can also be considered as effective targets for optical imaging and suggesting potential extensions to other metabolic indicators, such as hypoxia or redox potentials.

With regard to enzymatic targets, proteases have been among the most common enzymes in translational optical imaging. One of the main reasons for this selection is that proteolytic enzymes can be generically targeted using FRET-based and fluorogenic probes, where specific peptide substrates can be readily modified with fluorophore:quencher pairs or with fluorogenic dyes, respectively. In the context of cancer detection, cathepsins have been reported to be overexpressed in tumor cells^42^ and in tumor-associated macrophages.^43^ Cathepsins are involved in the degradation of extracellular matrix and are known to facilitate the growth, invasion, and metastasis of tumor cells.^44^ These features were exploited to design the cathepsin-targeting probe LUM015 in a first-in-human phase 1 trial in 15 patients undergoing surgery for soft tissue sarcoma and breast cancer (NCT01626066).^21^ LUM015 was injected intravenously before surgery, and imaging of resected human tissues showed significantly higher fluorescence in tumors vs normal tissues. Interestingly, the authors ran in parallel some preclinical studies in mice, where flow cytometry analysis showed that most fluorescently labeled cells were of tumor origin, with less than 10% being tumor-associated monocytes and macrophages.

Another example of cathepsin-activatable agents for in-human imaging of cancer cells was reported with the VGT-309 probe (Figure 2b).^24^ In this case, the FRET probe considered ICG as the fluorophore and QC-1 as the quencher. First, a clinical feasibility study was conducted during pulmonary tumor resection in canines, which was followed by a phase 1 safety study in humans (ACTRN12620000948998) and a phase 2 where the probe proved useful to detect some occult and nonpalpable pulmonary tumors during resection in real time (ACTRN12621000301864). Other than cathepsins, other enzymes have targeted for imaging of tumors in humans include PARP1, an enzyme associated with DNA damage response that is overexpressed in tumors due to the proliferation, mutational burden, and genomic instability of cancer cells.^45^ PARPi-FL was developed as a fluorescent topical probe for the early diagnosis of oral cancer and tested in 12 patients with histologically proven oral squamous cell carcinoma (OSCC) (NCT03085147). The patients gargled the agent for 60 s, followed by gargling a clearing solution for another minute, after which fluorescence signals were detected in the malignant lesions with sensitivity and specificity greater than 95%.

In addition to the microenvironment and enzyme-triggered fluorophores, protein receptors are the most common family of biological targets in translational optical imaging. Among these, FR-α has been widely targeted for cancer imaging (Figure 2c).^46^ FR-α are glycosylphosphatidylinositol-anchored membrane receptors that are overexpressed in solid tumors—including ovarian, breast and lung, among others—whereas their distribution in normal tissues is limited to low levels of expression on the surfaces of some organs (e.g., kidney, lung).^47^ Following the seminal report by van Dam and Ntziachristos on folate-FITC as a fluorescent probe for imaging FR-α receptors during surgery of 10 ovarian cancer patients (NTR1980; EudraCT 2009-010559-29),^10^ the same probe was used in 12 patients undergoing surgery for ovarian cancer and in 3 patients undergoing surgery for biopsy-proven FR-α-positive breast cancer (Figure 2c).^48^ Fluorescence imaging of ovarian tissue allowed the detection of lesions, including more than 10% that were not detected by visual inspection of palpation. On the other hand, in breast cancer surgery, the autofluorescence of normal breast tissue at 500 nm (e.g., emission maximum wavelength for FITC) interfered with the signal of the probe. This result prompted the chemical design of the NIR analogue OTL38, which retained the same folate moiety for targeting FR-α but replaced FITC with the S0456 fluorophore with emission maxima around 796 nm (Figure 2c). OTL-38 was used in 12 patients with ovarian cancer and accumulated in FR-α-positive tumors and metastases to facilitate the resection of 29% more malignant lesions that could not be identified by the surgeon. (2013-004774-10 and 2014-002352-12)^49^ Of note, subsequent studies have demonstrated the potential application of OTL-38 for the detection of pituitary adenomas (19 patients) (NCT02629549)^14^ and pulmonary adenocarcinomas (20 patients).^50^ A phase 3, multicenter study in suspected lung cancer patients was recently conducted to assess the efficacy of OTL-38 and NIR imaging to identify pulmonary nodules, as well as to assess the safety and tolerance of OTL-38 (112 patients) (NCT04241315).

Another family of surface receptors that has been commonly targeted for enhanced detection of cancer cells are growth factor receptors, including the epidermal growth factor receptor (EGFR) and the vascular endothelial growth factor receptor (VEGFR), which play important roles in tumor growth and cancer progression (Figure 2c).^51^ Unlike FR-α, these receptors have been most commonly targeted using fluorescently labeled antibodies. For instance, the anti-EGFR antibody cetuximab was conjugated to the NIR fluorophore IRDye800CW and used in 12 patients undergoing surgical resection of SCC arising in the head and neck (NCT01987375).^52^ Fluorescence intraoperative wide-field imaging was employed to differentiate between tumor and normal tissues with average tumor-to-background ratios over 5. In a parallel study, a NIR-labeled construct of the anti-VEGFR antibody bevazucimab was administered intravenously to 20 patients with primary invasive breast cancer and followed by fluorescence-guided surgery, where all but one tumor showed specific uptake (NCT02583568).^53^ These studies using NIR analogues of commercial antibodies will accelerate the efficient delineation of tumor margins using intraoperative imaging.

Although less common, other biological targets explored for imaging cancer cells include kinase receptors, among which tyrosine kinase c-Met was reported for the detection of colorectal cancer. c-Met is found on high levels on the surface of colorectal adenoma-carcinoma cells at early stages of the disease, potentially being a biomarker for the detection of neoplasia using fluorescence-guided endoscopy. Bearing this target in mind, the probe GE-137 (later known as EM-137) was developed as a high-affinity probe for c-Met.^16^ GE-137 was used in a first in-human pilot study demonstrating the safety of intravenous injection and its suitability for detection of neoplastic polyps, including some that were not detected by visible light. Follow-up studies with this probe have considered its application for detection of Barrett’s neoplasia, where it did not improve the outcome of surveillance endoscopies in a study with 15 patients (NCT03205501).^17^

Despite several successful examples of cancer-targeting probes and their potential application to improve clinical outcomes using fluorescence-guided intraoperative surgery, there is a growing need for additional biological targets in certain cancer types. For instance, the surface marker CD47 is found in a large proportion of bladder cancer cells and has been considered as a biomarker for solid bladder tumors.^54^ Aiming at this target, anti-CD47 antibodies modified with quantum dots emitting at 625 nm were designed as topical fluorescent probes for endoscopic imaging of human bladder cancer in combination with blue light cystoscopy.^55^

In the absence of defined molecular targets, combinatorial approaches can be considered to find binders of specific cancer cells. A good example of this approach involved the design of a fluorescent imaging probe for sessile serrated adenomas (SSAs), which are common in colorectal cancer but difficult to detect using white light microscopy.^56^ Because SSA cells display the V600E mutation in the BRAF gene, a phage-display library was generated to identify one hit peptide targeting colorectal cancer cells containing this mutation, and a subsequent fluorescent analogue was topically applied to 25 patients undergoing colonoscopies (NCT02156557). The fluorescent probe distinguished SSAs from normal mucosa with 89% sensitivity and 92% specificity, thus representing a good example of how early malignant lesions can be detected using fluorescence imaging in routine colonoscopies. In addition to applications in cancer cell detection, endoscopy-based imaging can also improve the prediction of therapeutic responses in patients with inflammatory bowel diseases. A notable example reported the generation of a fluorescent antibody for membrane-bound tumor necrosis factor (mTNF) and subsequent imaging in 25 patients with Crohn’s disease (NCT01275508).^57^ TNF-α has a critical role in the immunopathogenesis of Crohn’s disease, and several anti-TNF therapies have already been approved; however, there are few predictive biomarkers that can successfully identify responders over nonresponders. The topical administration of the mTNF-binding probe was able to improve stratification, with patients with high numbers of mTNF-positive cells showing shorter short-term response rates (92%) than patients with low amounts of mTNF-expressing cells (15%). These results highlight the potential of translational optical imaging as a technology not only to improve surgical outcomes but also to tailor therapeutic regimes and enhance personalized medicine.

## 3 Instrumentation, Image Acquisition, and Analysis

**Figure 3:**
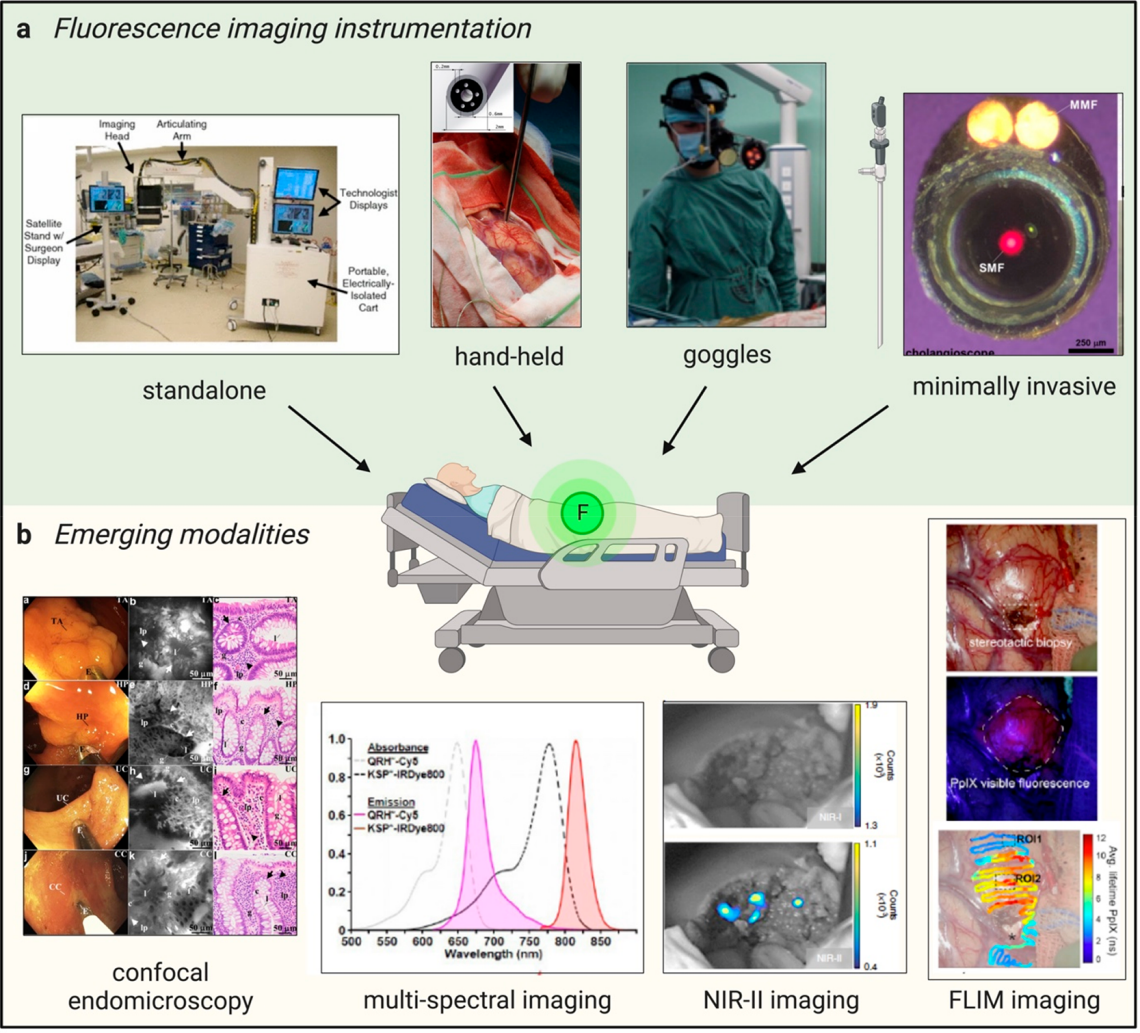
Maximizing the clinical impact of fluorophores. (a) Fluorescence imaging instrumentation used in the clinic. (Left to right) The FLARE imaging system deployed in the operating room.^59^ Reproduced from ref (59) with permission from Springer Nature. Hand-held device placed on a measurement site in a brain to detect PpIX fluorescence in patients with suspected glioblastoma.^65^ Reproduced from ref (65) with permission from Elsevier. Fluorescence goggles worn by a surgeon to image ICG in patients during hepatocellular carcinoma (HCC) resection.^67^ Reproduced from ref (67) with permission from Elsevier. Minimally invasive procedures with a graphical representation of a laparoscope and an en face view of a flexible NIR cholangioscope made of a single-mode fiber that delivers laser excitation and 2 multimode fibers (MMFs) that collect light.^72^ Reproduced from ref (72) with permission from Elsevier. (b) Emerging imaging modalities. (Left to right) Confocal endomicroscopy images of colonic mucosa in patients with IBD.^74^ Reproduced under a Creative Commons license from ref (74). Representative white light images (left panels), confocal endomicroscopy (middle panels), and histology images (right panels) are shown in decreasing order for adenoma, hyperplastic polyps, ulcerative colitis, and Crohn’s disease. Multispectral imaging featuring absorbance and emission spectra of the fluorescent peptides QRH*-Cy5 and KSP*-IRDye800 for multiplexed imaging in Barrett’s esophagus patients.^76^ Reproduced from ref (76) with permission from BMJ Publishing Group Ltd. NIR-II imaging of ICG in a HCC tumor after resection highlights no remaining signal left in the NIR-I window (top panel) but remaining malignant tissue in the NIR-II window (bottom panel).^77^ Reproduced from ref (77) with permission from Springer Nature. FLIM imaging of a superficial glioblastoma tumor using PpIX^78^ with white light image of the surgical field of view (top panel), standard fluorescence microscope image used for 5-ALA visualization (excitation 405 nm) (middle panel), and fluorescence lifetime image of the PpIX channel (629 nm/653 nm, bottom panel). Reproduced under a Creative Commons license from ref (78).

Fluorescence imaging was adapted for intraoperative guidance during surgery and diagnosis. To maximize the impact and benefit from recently developed chemical fluorophores, the design of suitable imaging systems is essential. Fluorescence imaging devices with lower sensitivity, red-shifted wavelengths, and more ergonomic features have been developed for use in the clinics over the past decade to provide clinicians with real-time visualization of tumors and lymph nodes, as well as other vital structures. In this section, we present an overview of different imaging devices that have been used in combination with fluorescent agents and the opportunities and challenges resulting from their application in clinical settings (Figure 3).

### Standalone Fluorescence Imaging Systems

Standalone fluorescence imaging systems are composed of three components: (1) an imaging head supported by (2) an articulating arm and (3) a central processing unit, which processes the fluorescence signals and displays the images on a monitor. One early example is the SPY fluorescent imaging system, which has been used for NIR angiography using ICG in patients with peripheral arterial disease (PAD) or vascular trauma^58^ and was one of the first systems to be approved by the FDA in 2005. These systems generally have a wide range of functionalities such as a large field of view (FOV) size and large working distance. In another system, the FLARE imaging head is capable of simultaneous, real-time image acquisition which allows surgeons to visualize the injection of ICG, monitor lymphatic flow, and pinpoint exactly the lymph node of interest in oncologic surgery.^59^ The main limitation of these standalone devices is that they mostly provide only a top-down FOV. As such, surgeons are expected to associate the image displayed on the monitor to the anatomic region of interest. The bulky size of these devices may also disrupt surgical workflow in the operating theater.

### Diffuse Optical Tomography (DOT)

The DOT technology builds on the design of the optical mammoscope. Briefly, it involves a platform with an adjustable cup where one breast is suspended for illumination with NIR light.^60^ DOT employs NIR excitation to assess optical properties within biological tissue and tracks spatial–temporal fluctuations in light absorption and scattering properties within the tissue to reconstruct spatial maps of optical properties. The DOT system, together with the administration of the nonspecific cyanine dye omocianine, facilitated the imaging of breast lesions.^61^ These signals were detectable through the DOT system and enabled the identification of 5 out of 10 malignant lesions in patients suspected of breast cancer.

### Hand-Held Devices

To overcome the limitations of limited portability and lack of flexibility of standalone devices, hand-held systems have been developed to provide better access to complex tissue geometries such as navigating around the head and neck or even inside tumors. For example, the fluorescence signals of the cathepsin-activatable LUM015 were detected with a sterile hand-held device that was inserted in the surgical cavity, enabling the detection of tumors with direct display on a computer monitor.^62^ In another system, Artemis is an easily maneuverable hand-held system that can image liver metastases from colon tumors.^63^ To reduce the high cost of large imaging devices, research groups are developing cheaper, portable modular intraoperative fluorescence imaging devices for fluorescence-guided surgery. One example is the Small Portable Interchangeable Imager of Fluorescence (SPIIF), which can be acquired for several thousand dollars.^64^ Hand-held probes can also function as standalone devices where they can be coupled to any intraoperative navigation system or be used with a fluorescence-guided resection surgical microscope.^65^

### Fluorescence Goggles

Fluorescence goggles display real-time visualization to surgeons via head-mounted displays. This can be achieved by incorporating miniaturized imaging detectors, powerful processors, and fast image-processing units into a pair of wearable goggles. These goggles allow surgeons to look at surgical sites instead of images on a separate display unit. Furthermore, in contrast to hand-held probes, the illumination module is mounted onto the head-mounted display, leaving clinicians hands-free with improved workflow. Several studies have demonstrated the use of fluorescence goggles with ICG during intraoperative imaging.^66,67^ Recently developed goggles allow for dual-mode imaging, autofocus, and autocontrast adjustment.^68^ A wearable goggle augmented imaging and navigation system (GAINS) can project both NIR fluorescence and white light images onto a head-mounted display and has been used to identify sentinel lymph nodes in patients with breast cancer and melanoma.^69,70^

### Minimally Invasive Procedures

To reduce invasion and morbidity associated with surgical procedures, minimally invasive methods, such as robotic and laparoscopic surgery, have been on the rise. These systems allow clinicians to visualize and remove suspected lesions with minimal intervention. Several laparoscopic systems are commercially available, and they can be equipped with both NIR imaging and regular white light imaging modules. For instance, ICG fluorescence emission was used to guide laparoscopic and robot-assisted resections of colorectal liver metastases in patients.^71^ A flexible cholangioscope was recently reported to visualize the NIR fluorescence emission of EGFR and ErbB2 receptors following administration of a fluorescent probe in the human esophagus to identify Barrett’s neoplasia in human bile ducts.^72^

### Imaging Modalities and Opportunities

#### Confocal Endomicroscopy

With the incorporation of confocal imaging into minimally invasive procedures, fluorescence confocal endomicroscopy is a promising technique for deep *in vivo* imaging of tissues, where high-resolution cross-sectional images can be achieved at the micrometer scale. Dual-axis NIR fluorescence endoscopes have been developed to obtain clinical NIR images of human colorectal cancer in the colon of human patients.^73^ This technology also offers subcellular resolution to provide *in vivo* images with “histology-like” quality in real time; therefore, it can assist physicians in making clinical decisions and prevent patients from potentially undergoing biopsy procedures. An advanced distal MEMS scanner has been used for real-time histopathology where crypt lumens and lamina propria could be distinguished.^74^ This work demonstrates the potential of confocal endomicroscopy for future routine medical endoscopy linked to disease diagnosis and therapy monitoring.

#### Multispectral Imaging

Using fluorophores with minimal overlap between absorbance and emission spectra can be used to visualize multiple targets. This feature can lead to safer surgeries, where several targets and structures must be detected. For instance, both MB and ICG have been used simultaneously during colorectal surgery, whereas the former was used for the ureter, while the latter enabled detection angiography for anastomotic perfusion evaluation. The combination of the two fluorophores was utilized to protect and preserve their key structures, leading to better surgical outcomes.^75^ Another interesting example was the simultaneous detection of premalignant lesions with two nonoverlapping fluorescently labeled heptapeptides, specific for transmembrane tyrosine kinase receptors EGFR and ErbB2, in Barrett’s esophagus patients.^76^

#### The NIR-II Window

The NIR-II imaging window (1000–1700 nm) offers highly desirable properties for translational imaging, such as reduced tissue autofluorescence and minimal photon scattering, thus improving the quality of images substantially. Although there are currently no clinically approved NIR-II imaging agents to date, it was recently demonstrated that an image-guided resection procedure during human liver tumor surgery that detected ICG in the NIR-II window could delineate lesions that were missed by NIR-I imaging.^77^

#### Fluorescence Lifetime Imaging Microscopy (FLIM)

FLIM is an attractive imaging modality, as it can distinguish fluorophores with overlapping spectral properties based on their fluorescence lifetimes. PpIX displays fluorescence lifetimes in the low nanosecond range when excited with near-UV light. Using 355 nm excitation to simultaneously excite both PpIX and the metabolic biomarker nicotinamide adenine (phosphate) dinucleotide (NAD(P)H), intraoperative FLIM could simultaneously detect the emission of PpIX and NAD(P)H from patients *in vivo* during craniotomy procedures.^78^ This study demonstrates that an intraoperative FLIM approach can serve as a clinical tool to identify tumor areas for resection as well as a research tool to study microenvironmental changes in tumors *in vivo*.

#### Algorithms and Decision Making

Key surgical decisions and diagnosis are traditionally made by human visual judgment; however, the interpretation of fluorescence signals may not always be straightforward because the intrinsic photophysical properties of a fluorophore may change according to the environment. Fluorescence emission *in vivo* does not remain static, and maximum contrast does not always exist between malignant and healthy tissues. In addition, there are also other confounding factors such as photobleaching, pharmacokinetic clearance, fluorophore concentration, or even variation in the excitation intensity from different sources. To overcome these challenges and help with clinical fluorescence analysis, mathematical and computer algorithms have been developed.^79^ One example of these approaches has been the design of mathematical algorithms that account for the variation of the photophysical properties of environmentally sensitive fluorophores. For instance, the FDA-approved dye fluorescein exists as an equilibrium mixture of four species (i.e., cation, neutral, anion, and dianion) depending on the environmental pH. This pH sensitivity was exploited to detect dental biofilms using a spectral unmixing algorithm.^80^ Fluorescence image guidance also holds great potential to accelerate the tissue identification and delineation of malignancy. In the past few years, several groups have attempted to incorporate artificial intelligence (AI) in fluorescence-guided surgeries to interpret fluorescence signals more objectively,^81−83^ and computer algorithms have enabled discrimination between tissue types thanks to the differential perfusion patterns of ICG in cancerous, benign, and normal tissues.^83^

## Conclusions and Outlook

Optical agents for molecular imaging are the backbone of fluorescence-guided treatments. An ideal contrast agent should bind exclusively to a disease-specific biomarker, accumulate sufficiently in the target area, display high signal-to-background ratios, and be rapidly excreted from nontarget tissues. Recent advancements in the development of molecular optical agents have focused on the modification of existing dyes to improve the penetration depth and signal-to-background ratios. This includes dyes in the NIR window and structural modifications of existing scaffolds toward longer absorbance and emission wavelengths. There has also been increasing research on fluorescent structures to image vital structures (e.g., nerves).^84,85^ These fluorophores hold potential not only to improve the outcomes of tumor resection but also to facilitate the visualization of anatomical structures in challenging surgeries.

Even though significant advancement has been achieved in the past decade, there are only a few probes that have been translated to clinical environments, largely due to regulatory issues. As more molecular optical agents are developed, the recently explored dyes and targeting moieties will require further clinical assessment to address potential safety and toxicity concerns. Another barrier for translation is clinical adoption and the learning curve for clinicians to be comfortable with using optical agents for intraoperative imaging. For instance, this includes the implementation of such technologies in a clinical setting and the correct interpretation of fluorescence readouts. Despite these challenges, targeted fluorophores hold clear potential for use in translational optical imaging.

In parallel, the development of imaging devices has also positively contributed to the translation of fluorescent probes for use in humans. Advances in the engineering of better, more ergonomic, and less invasive imaging devices have facilitated the use of optical imaging agents in a clinical environment. Although such progress is notable, more efforts need to be done to design devices with improved optical resolution (e.g., lower background, multiplexing capabilities) that can accelerate the translation of imaging modalities, such as confocal endomicroscopy and multispectral imaging. Developments in these areas will allow for the improved usage of imaging devices and optical agents.

To better interpret the fluorescence signals from targeted probes, data analysis and machine learning must be integrated into imaging workflows and dynamically monitor the signals from fluorophores *in vivo*. The implementation of AI as a real-time decision-making tool holds great potential for both diagnosis and therapy. Currently, many algorithms are limited in terms of the range of fluorophores and biological targets. This limitation hinders broad application to a wide range of use cases, but the adaptation of existing software will expand the scope of current data analysis tools and allow integration in surgical workflows to help clinicians interpret fluorescent signals objectively. In summary, translational optical imaging is full of opportunities for chemists, molecular and cell biologists, engineers, data scientists, and clinicians. Progress in the development and optimization of optical probes and imaging devices will yield the next generation of imaging technologies and clinical modalities to improve the efficiency and accuracy of clinical procedures.
